# Talking to patients about diabetes and eye health

**Published:** 2015

Diabetes is a complex disease requiring the involvement of many health professionals. As a result, people with diabetes are faced with multiple appointments and multiple health messages to understand and make sense of. For example, blood sugar levels (and what is ideal), foot health (and warning signs), kidney checks and eye examinations.

As eye care practitioners, we are faced with the challenge of trying to talk to patients – who are often without symptoms – about their vision in relation to their diabetes. We must encourage and support them to return for eye examinations and treatment (see page 68).

Whenever diabetes patients come into our eye clinics, we can also support their overall health and diabetes treatment by offering suitable information, education and support. This is important as good diabetes control helps to slow down the progression of diabetic eye disease. Here are a few practical things we can do.

- Send regular reminders to other health professionals in the hospital or community to advise patients with diabetes about the importance of annual eye examinations and where to go.
- Discuss the variety of diabetes checks and clinics the patient attends and how often. Encourage patients to keep their appointments and stay in contact with colleagues in other departments, so you can give patients all the information they need. This will help them to obtain and keep appointments.
- Educate patients about how to stay healthy and manage their diabetes (see panel below). This slows down the progression of diabetic eye disease. Even if patients have heard this elsewhere, change takes time, and the education (and encouragement) needs to be ongoing. Educate patients about their individual situation. Use their own retinal images to show them any changes (without blaming them!) or use the poster on pages 70–71. The far right-hand column in the poster contains suggestions for how to talk to patients about what is going on, and what is likely to happen next.

**‘Making lifestyle changes is difficult. We must empower patients with information and support’**

Making lifestyle changes is difficult. We must empower patients with information and support so they can take an active role in their own health-including coming for follow-up appointments and treatment. We can also lead by example by ensuring that we follow the advice below ourselves.

With thanks to Tunde Peto and Peter Blows

