# Supplementary material for: Talking to patients about diabetes and eye health
Source: Community Eye Health. 2015;28(92):74. (PMC4944100)
Supplement: Related texts

# Brief report: Information for patients: living with diabetes and protecting your eyes

**Published:** 2015

**Authors:** Zhara Gregory

**Affiliations:** Diabetes Specialist Nurse: Homerton Diabetes Centre, London, UK. **zhara.gregory@nhs.net**

**Figure:** Zhara Gregory

In order to protect you reyes from being damaged by diabetes, there are three things you must aim for:

1. Stable blood glucose (blood sugar).
2. Healthy blood pressure, achieved by exercising and taking any prescribed blood pressure medication.
3. A healthy weight and low cholesterol: Achieved by a combination of eating well and exercising.

All these can be achieved by taking your medication as prescribed, by following a healthy diet and by taking regular exercise. Follow the tips below to make changes to your habits. Maybe start with one change a week! This will help to protect you reyes until your next visit.

## Medication

Take your medication regularly. Set an alarm (e.g. using a phone) asa reminder, or ask a family member to help remind you.

Eat something just before taking your medication as this will reduce the likelihood of having an upset stomach.

## Eating and drinking

In order to keep blood glucose levels as stable as possible, pay attention to foods that affect your blood glucose – e.g. fruit, fruit juice, potatoes, rice, and so on.

- Eat three regular meals a day. Missinga meal, or doing more exercise than usual, can result in hypoglycaemic episodes (low blood glucose levels). This is characterised by shaking, pounding heart, nervousness, sweating, tingling, and hunger. If left untreated, it can lead to unconsciousness.
- Plan meals that look like the plate in Figure 1: ½ a plate should contain vegetables (i.e., what you can hold in two hands), ¼ plate should contain starchy foods (i.e. the size of your fist) and ¼ plate should hold protein (i.e. the size of the palm of your hand).
- Some starchy foods/carbohydrates break down more slowly than others, and are therefore better for keeping your blood sugar stable. Where available, choose wholegrain or brown bread rather than white, whole potatoes rather than mashed, yams/sweet potatoes rather than ordinary potatoes.
- Eat a maximum of three portions of fruit a day – each portion at a different time of the day.
- Don't drink more than one glass of fruit juice per day as they are full of sugars.
- Avoid sugary drinks, e.g. cola – drink plenty of water instead.
- Avoid alcohol as it makes hypoglycaemia more likely to occur. Alcohol can raise blood pressure and lead to weight gain.
- Sweets, biscuits and cakes can be eaten once in a while as a treat, i.e. once a week.
- Avoid eating visible fat (cut it off the meat before you eat it)
- Avoid fried foods – steamed or baked is better.

**Figure 1.** A healthy plate

## Exercise

- Exercise helps to lower blood pressure, glucose and cholesterol levels, improves energy levels and general well being, and promotes weight loss. Exercising for 30–50 minutes at least 4–5 times a week is best, but you can start with as little as 20 minutes 3 times a week and build it up from there. Ask for advice from a doctor.
